# Optimization of amino acid replacement costs by mutational pressure in bacterial genomes

**DOI:** 10.1038/s41598-017-01130-7

**Published:** 2017-04-21

**Authors:** Paweł Błażej, Dorota Mackiewicz, Małgorzata Grabińska, Małgorzata Wnętrzak, Paweł Mackiewicz

**Affiliations:** Department of Genomics, Faculty of Biotechnology, University of Wrocław, ul. Joliot-Curie 14a, 50-383 Wrocław, Poland

## Abstract

Mutations are considered a spontaneous and random process, which is important component of evolution because it generates genetic variation. On the other hand, mutations are deleterious leading to non-functional genes and energetically costly repairs. Therefore, one can expect that the mutational pressure is optimized to simultaneously generate genetic diversity and preserve genetic information. To check if empirical mutational pressures are optimized in these ways, we compared matrices of nucleotide mutation rates derived from bacterial genomes with their best possible alternatives that minimized or maximized costs of amino acid replacements associated with differences in their physicochemical properties (e.g. hydropathy and polarity). It should be noted that the studied empirical nucleotide substitution matrices and the costs of amino acid replacements are independent because these matrices were derived from sites free of selection on amino acid properties and the amino acid costs assumed only amino acid physicochemical properties without any information about mutation at the nucleotide level. Obtained results indicate that the empirical mutational matrices show a tendency to minimize costs of amino acid replacements. It implies that bacterial mutational pressures can evolve to decrease consequences of amino acid substitutions. However, the optimization is not full, which enables generation of some genetic variability.

## Introduction

Biological evolution is driven by changes in genetic material, which generates variation between organisms. Many of these changes result from substitutions of one nucleotide for another and occur mainly during replication of DNA, when two strands, called leading and lagging are synthesized by different mechanisms^1, 2^. The process demands temporal separation of double stranded DNA into two single strands. In such single-stranded state, spontaneous deamination of C and A are common mutations. In particular, the deamination of C to U or 5-methylcytosine to T occurs more frequent than in double-stranded DNA^3, 4^. The probability of such mutations is higher on the leading strand because this strand stays longer in the single state, as a template for synthesis of the lagging strand^5–7^. The cytosine deamination on the lagging strand template result in C → T mutations on leading strand. Therefore, the DNA strands are characterized by different patterns of nucleotide substitutions^1, 2, 5, 8^. In consequence, the leading strand becomes more rich in guanine than cytosine and, to a lesser extent it receives more thymine than adenine in comparison to the lagging strand in many bacterial genomes^9–11^. The characteristic asymmetry in nucleotide composition occurs between these differently replicated strands not only in majority bacterial genomes^8, 12–20^ but also eukaryotic genomes^21–25^.

The ‘asymmetric’ mutational pressures influence also evolutionary rate of genes located on the DNA strands^26–29^. The lagging strand genes show generally a larger substitution rate than the leading strand genes, and homologs lying on differently replicated DNA strands are characterized by higher divergence than those staying on the same type of strands. The difference in the rate of nucleotide substitutions between the strands was shown in the experimental study of *Bacillus subtilis* ^30^, in which the rate of point mutations in core genes on the lagging strand appeared higher than on the leading strand. The differences were most pronounced in non-synonymous substitutions. The ‘asymmetric’ structure of bacterial chromosomes is also associated with symmetric genomic inversions containing the origin of replication^31–33^, a bias in gene translocations between the lagging to leading strands and stability of gene positions in chromosome^34–38^ as well as a preference in location of essential genes in the leading strand^39, 40^.

Such spontaneous mutations introduced during DNA replication into protein-coding sequences are deleterious, when they cause replacements of amino acids with different physicochemical properties leading to non-functional products. Repairing of mutations is also energetically costly for organisms^41, 42^. Thus, it seems that minimization of mutational pressure and its cost should be favoured during evolution. Actually, it was postulated that the mutational pressure and the genetic code are optimized to minimize harmful effects of mutations on protein-coding sequences and translation errors as a result of their coevolution^43–52^. However, recent analyses about the optimization of the genetic code showed that there exist alternative genetic codes that are much better optimized in respect to the polarity than the canonical one^53^. Moreover, current knowledge about the genetic code origin and evolution indicates that biosynthetic relationships between amino acids were the main factor that structured the genetic code, whereas the physicochemical properties of amino acids played only a subsidiary role in its evolution^54, 55^.

On the other hand, mutations are essential for evolution because they deliver the raw material of genetic variation. They can turn out beneficial especially for organisms living in rapidly changing environments. In such habitats the increase in mutation rate is favoured because it enriches the genetic variation and enables quick adaptation of the organisms to the new conditions^56–58^. As a result, a trade-off between the necessity to preserve accurate genetic information and requirements for adaptational flexibility of organisms would be observed. It should lead to some kind of optimality of the mutation process and evolution of mutation rate in organisms^59–62^. However, not only the global mutation rate but also relative rates of nucleotide substitutions can be subjected to this optimization^63, 64^. For example, we can expect that some mechanisms associated with replication of DNA and its repairing evolved to minimize probability of spontaneous point mutations that cause replacements of amino acids with disparate physicochemical properties, e.g. hydropathy or polarity. To verify hypotheses if mutational pressures operating in various bacterial genomes are random or they are optimized in respect of amino acid replacements in products of protein-coding genes, we compared the empirical mutational pressures derived from bacterial genomes with their best possible alternatives that minimized or maximized costs of amino acid replacements.

## Results

### Comparison of matrices according to costs of amino acid replacements

The aim of the study was to assess to what extent bacterial nucleotide mutational pressures are optimized to minimize or maximize non-synonymous substitutions in protein-coding sequences resulting in amino acid replacements and changes in their physicochemical properties. We have focused particularly on hydrophobic^65^ and polar properties^66^, which are important characteristics of proteins. The pressures were described by mutational probability matrices containing probabilities of all possible twelve nucleotide point mutations. To check the optimization level of the empirical mutational matrices, we compared their effect with that of theoretical probability matrices that produced the same nucleotide stationary distribution as the corresponding empirical matrices, and minimized or maximized the costs of the amino acid replacements. Thus, these optimized matrices represented possible boundary reference states to which the natural pressures can evolve.

**Figure 1:**
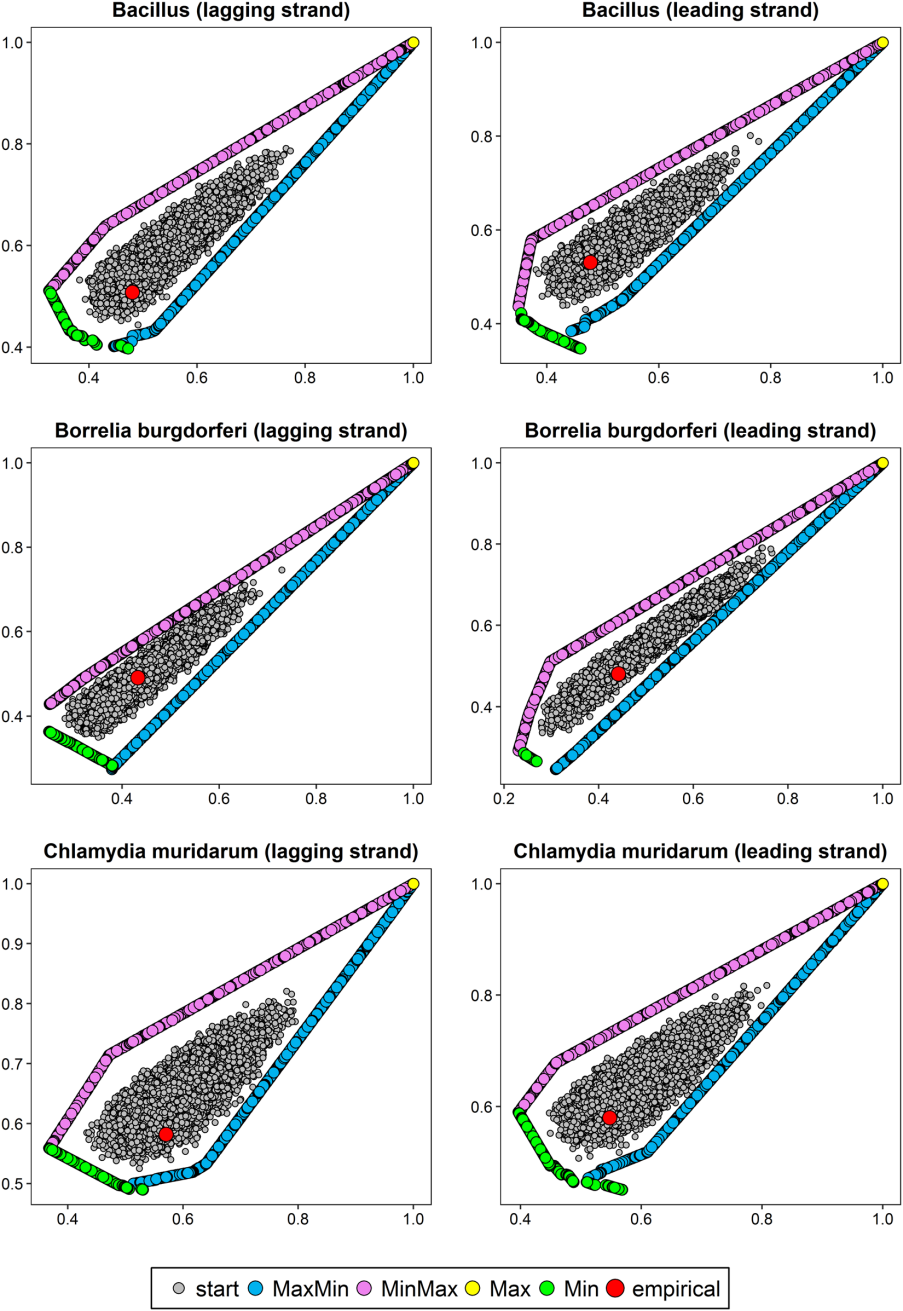
Comparison of costs of amino acid replacements in two physicochemical properties, hydropathy (x-axis) and polarity (y-axis) generated by: random started matrices (start), empirical matrices (empirical) and matrices maximizing hydropathy and minimizing polarity (MaxMin); minimizing hydropathy and maximizing polarity (MinMax) as well as maximizing (Max) or minimizing (Min) the both costs.

**Figure 2:**
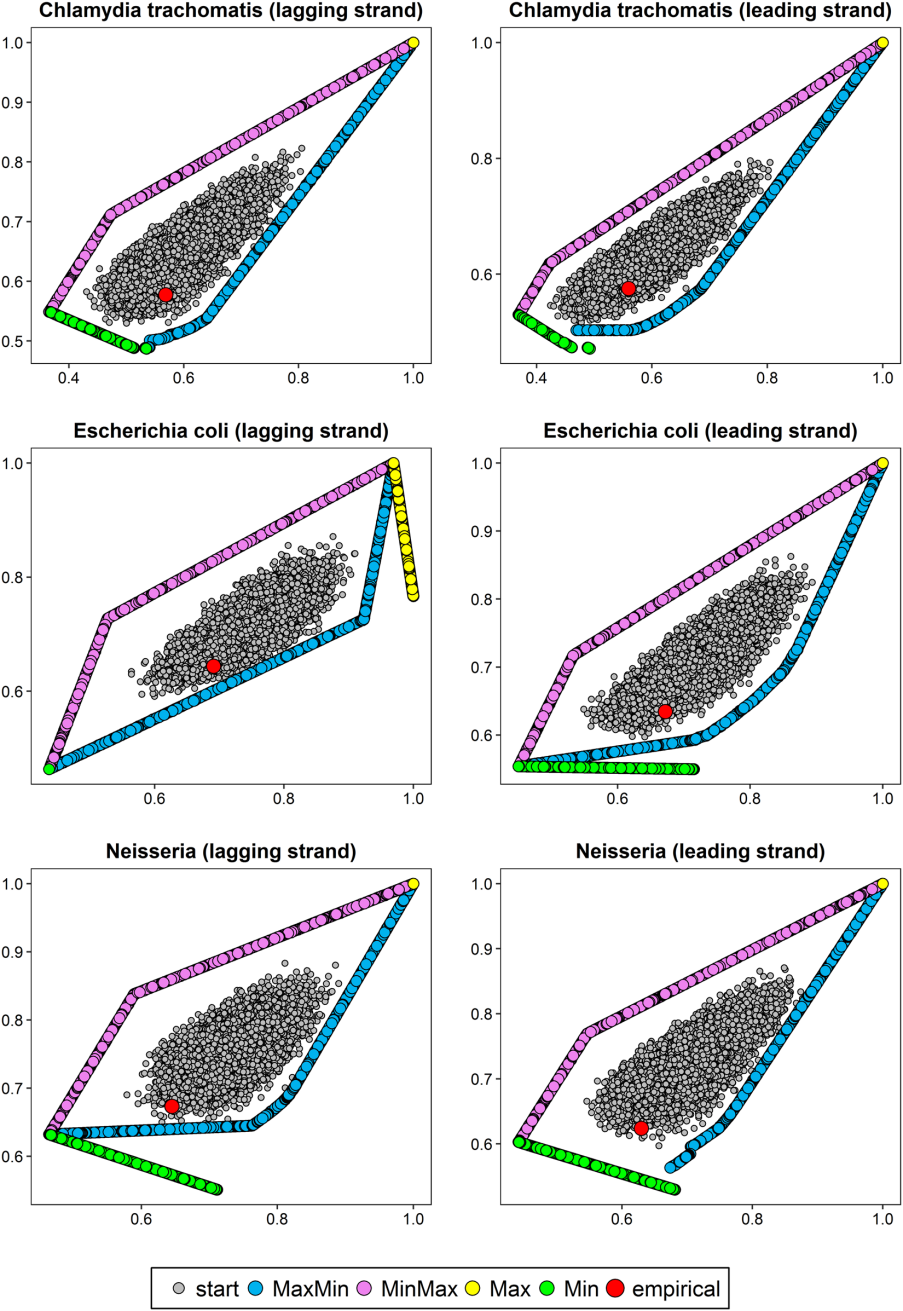
As in Fig. 1.

**Figure 3:**
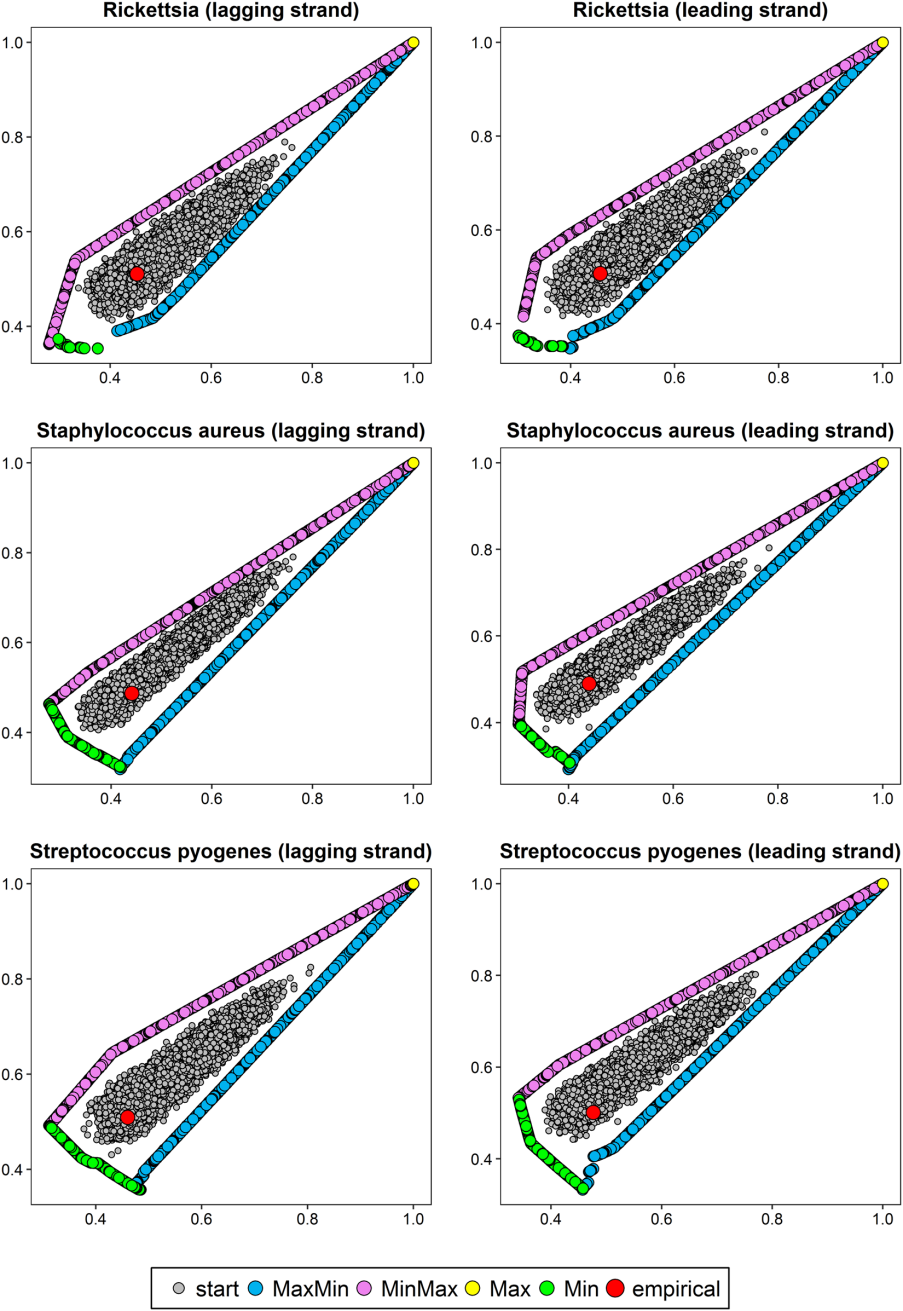
As in Fig. 1.

The optimization level was tested on protein-coding sequences (described by codon frequencies) from nine bacterial genomes (Table S1). The sequences were extracted separately from differently replicated DNA strands (leading and lagging) because they are characterized by different mutational patterns. We considered four scenarios of optimization: matrices maximizing hydropathy and minimizing polarity (MaxMin); minimizing hydropathy and maximizing polarity (MinMax); maximizing (Max) or minimizing (Min) the both costs. Since we optimized matrices according to two physicochemical properties simultaneously, we received sets of matrices called Pareto sets, i.e. non-dominated solutions such that none of the studied physicochemical property can be improved in value without degrading the other property. The obtained Pareto fronts of optimized matrices with starting and empirical matrices computed for differently replicated DNA strands of individual genomes are shown in Figs 1, 2 and 3. The x and y axes represent costs of amino acid substitutions according to hydropathy and polarity, respectively, normalized by the maximum found cost.

The centre of the plots is occupied by randomly generated starting matrices. The points are surrounded by two clear Pareto fronts for the MaxMin and MinMax scenarios, in which the algorithm found mutational matrices minimizing one type of amino acid replacement costs and maximizing the other. In some cases (e.g. *Borrelia burgdorferi* or *Staphylococcus aureus* lagging DNA strand), the matrices are arranged in single straight lines with a positive slope (Figs 1 and 3), whereas in others (e.g. *Chlamydia trachomatis* or *Escherichia coli* lagging DNA strand), the lines bend at one or two points (Fig. 2).

The MaxMin and MinMax Pareto fronts converge at the large values of costs (the upper right part of the plots) to the matrices that maximized the costs of amino acid replacements in both properties (Max scenario). Although the algorithm found such matrices in the number from 93 to 4134 (in dependence on the assumed stationary distribution), they were very similar in their nucleotide transition probability rates and generated costs. As a consequence, they are visible as one point in the scale of the plots (Figs 1, 2 and 3). The only exception is the case of *Escherichia coli* lagging DNA strand, where the maximized matrices create a linear Pareto front with negatively correlated costs of the two physicochemical parameters (Fig. 2). On the other hand, the matrices that minimized the costs (Min scenario) are located in the lower left part of the plots (Figs 1, 2 and 3). With the exception to the case of *Escherichia coli* lagging DNA strand, where the matrices are visualized by one point in plots (Fig. 2), in other cases they create a Pareto fronts, usually with a linear course with a negative slope.

The most interesting is the position of empirical mutational matrices in the space of the two costs. They are usually located at the edge or lower left part of the distribution of starting matrices and close to Pareto fronts of scenarios, in which at least one cost of amino acid replacements was minimized (Min, MaxMin or MinMax) (Figs 1, 2 and 3). Simultaneously, the empirical matrices are located far from the matrices maximizing the costs of amino acid replacements.

To objectively compare the location of various empirical matrices to the Pareto fronts, we calculated relative minimal distances to these fronts *r*_*i*_ for the individual scenarios (Table 1). The distance measures a relative difference in the costs associated with replacements of amino acid with different physicochemical properties generated by empirical matrices in comparison to the matrices optimized under these costs. The smaller value, the more similar costs generated by the empirical matrix in comparison to the matrices from the corresponding Pareto front. The results demonstrate that the smallest distances of the empirical matrices were usually to Pareto fronts obtained under the scenario MaxMin and in one case to the MinMax front (*Borrelia burgdorferi*, lagging strand). The smallest distance (0.0449) showed the lagging strand matrix from *Neisseria* to the Pareto front of matrices MaxMin optimized.

**Table 1:** Relative minimal distances of empirical matrices from two DNA strands in bacterial genomes to respective Pareto fronts of matrices maximizing hydropathy and minimizing polarity (MaxMin); minimizing hydropathy and maximizing polarity (MinMax); maximizing (Max) and minimizing (Min) the both costs.

Genome	Leading strand				Lagging strand			
	Max	MaxMin	MinMax	Min	Max	MaxMin	MinMax	Min
*Bacillus* species	0.662	0.090	0.093	0.155	0.700	0.078	0.118	0.104
*Borrelia burgdorferi*	0.632	0.052	0.088	0.229	0.674	0.087	0.058	0.181
*Chlamydia muridarum*	0.650	0.086	0.136	0.128	0.648	0.076	0.167	0.109
*Chlamydia trachomatis*	0.667	0.077	0.122	0.135	0.656	0.073	0.166	0.105
*Escherichia coli*	0.642	0.062	0.187	0.110	0.396	0.045	0.187	0.371
*Neisseria* species	0.639	0.091	0.179	0.091	0.649	0.045	0.178	0.128
*Rickettsia* species	0.674	0.075	0.093	0.157	0.673	0.080	0.086	0.161
*Staphylococcus aureus*	0.687	0.081	0.086	0.146	0.699	0.080	0.082	0.140
*Streptococcus pyogenes*	0.693	0.075	0.112	0.120	0.699	0.093	0.101	0.106

The distances were calculated in the final 2000th step of simulations.

On average, the empirical matrices were located much closer to matrices that minimized at least one parameter (*r*_*MaxMin*_ = 0.075, *r*_*MinMax*_ = 0.124 and *r*_*Min*_ = 0.149) than to matrices that maximized two costs (*r*_*Max*_ = 0.652). The differences between the distances (*r*_*Max*_ vs others) were statistically significant (p_BH_ < 0.0003, Wilcoxon test with Benjamini-Hochberg correction for multiple testing). Significantly smaller differences were also for distances of the empirical matrices to MaxMin optimized matrices than to MinMax optimized matrices (p_BH_ < 0.0008) and matrices minimizing two costs (p_BH_ < 0.0003). However, the distances of the empirical matrices to those produced under Min scenario were not significantly different (p_BH_ = 0.29) when compared with the distance to the Pareto fronts obtained in the MinMax scenario. Considering two extreme cases in which both costs were minimized (Min scenario) or maximized (Max scenario), each empirical matrix was closer to the Pareto fronts created by the matrices minimizing both parameters than those maximizing the costs. The average distance of the empirical matrices to the Pareto front of the minimizing matrices was almost five times smaller than to the maximizing matrices.

The empirical matrices from the leading DNA strand were slightly closer to the Pareto fronts of matrices that minimized two costs than the lagging strand matrices (mean 0.141 vs 0.156). Simultaneously, the leading rather than lagging strand matrices were more distant from the matrices maximizing two parameters (mean 0.661 vs 0.644). However, these differences (and also others for any scenarios) were not statistically significant in respect to the DNA strands (p_BH_ > 0.79).

To check universality of our findings, we carried out similar analyses based on other indices and scoring matrices describing various physicochemical properties of amino acids: conformational parameter for alpha helix and beta-sheet^67^, Grantham’s chemical distance^68^, Miyata’s amino acid pair distance^69^ and Mohana’s EMPAR matrix^70^. In total, 18 mutational matrices (from 9 genomes and 2 DNA strands) were tested under 21 pairwise combinations of 7 physicochemical properties, which gave 378 cases.

**Figure 4:**
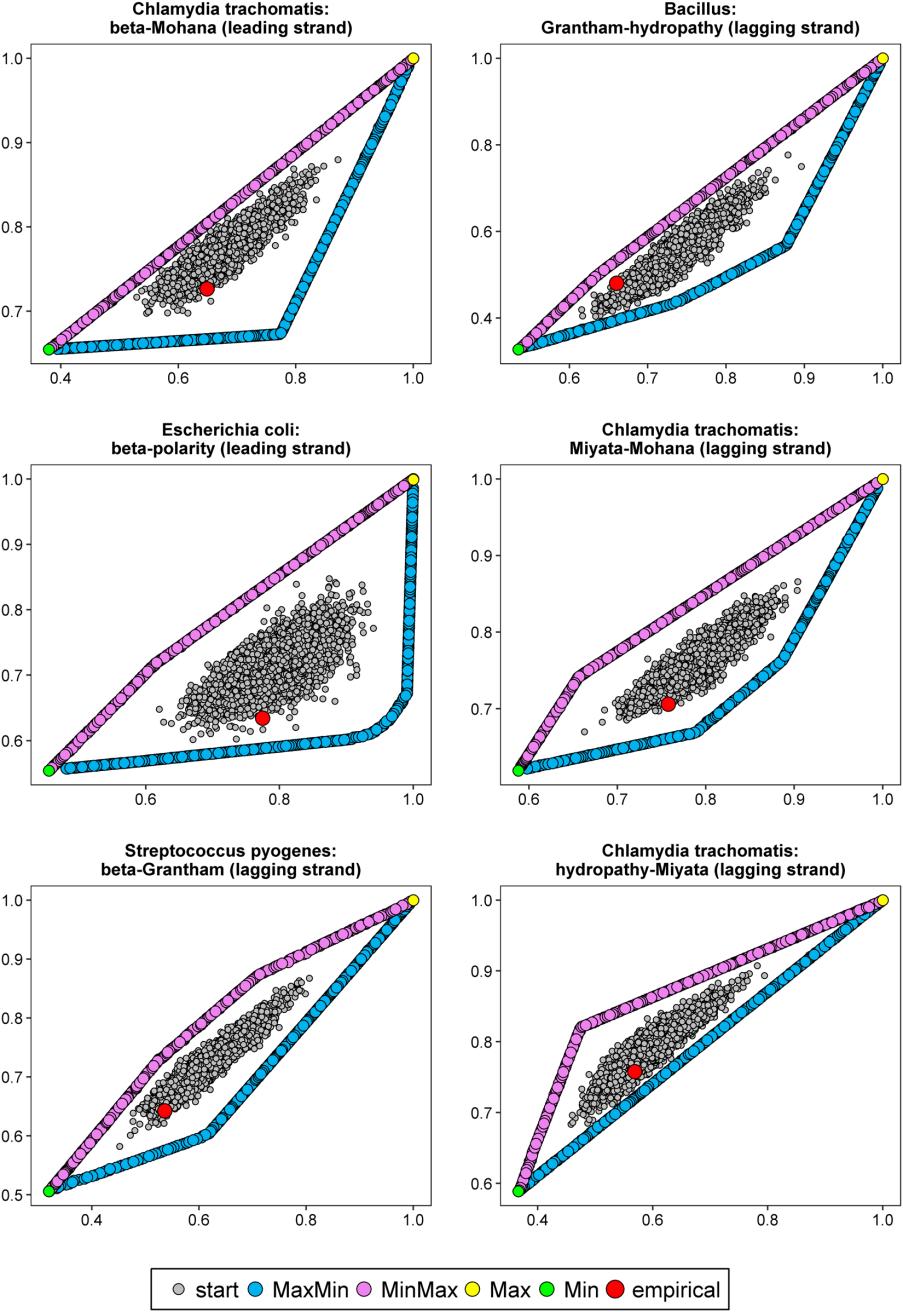
Comparison of costs of amino acid replacements in two selected physicochemical properties, generated by: random started matrices (start), empirical matrices (empirical), matrices maximizing one and minimizing other property (MaxMin) and *vice versa* (MinMax) as well as matrices maximizing (Max) or minimizing (Min) the both costs.

**Figure 5:**
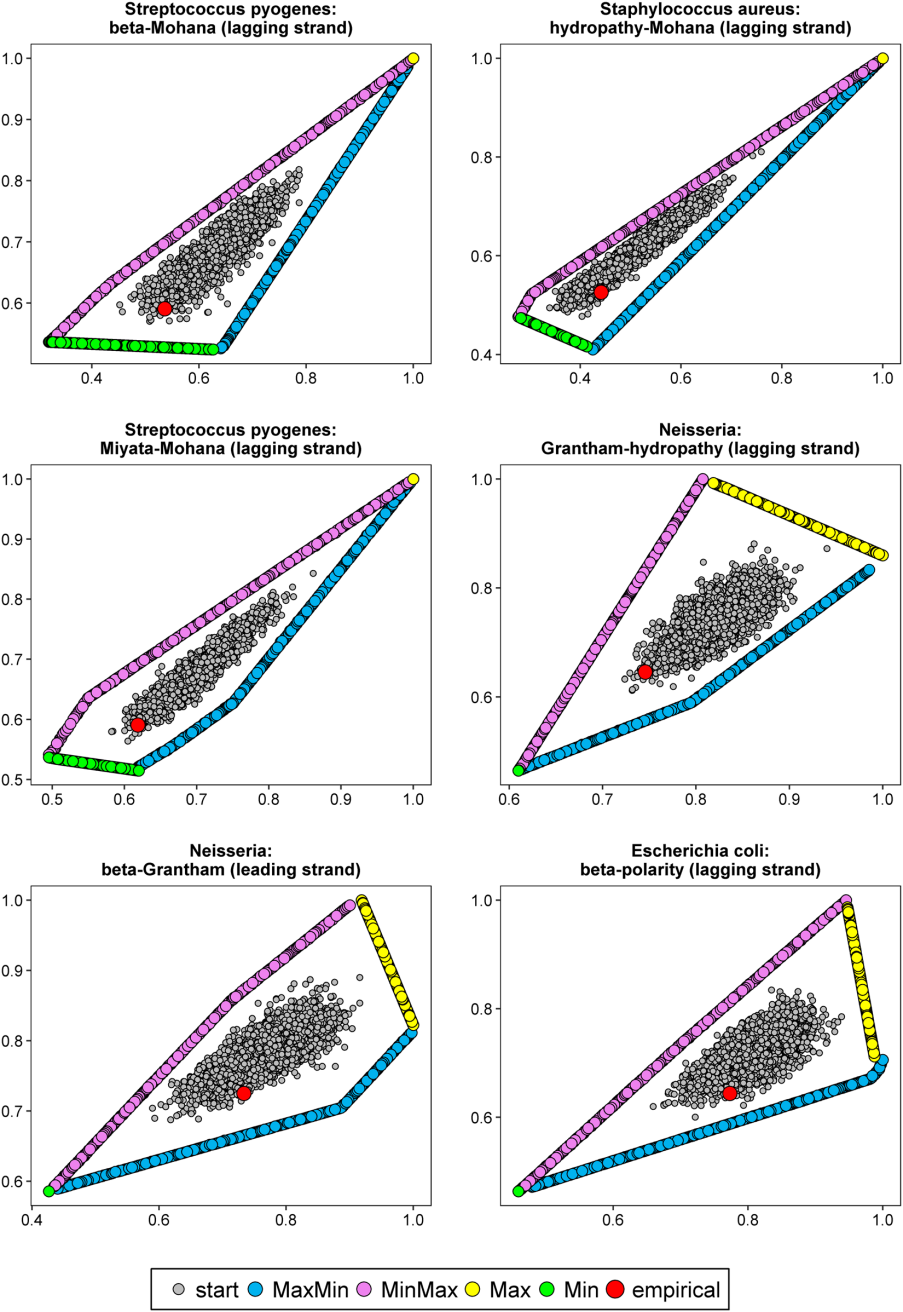
As in Fig. 4.

**Figure 6:**
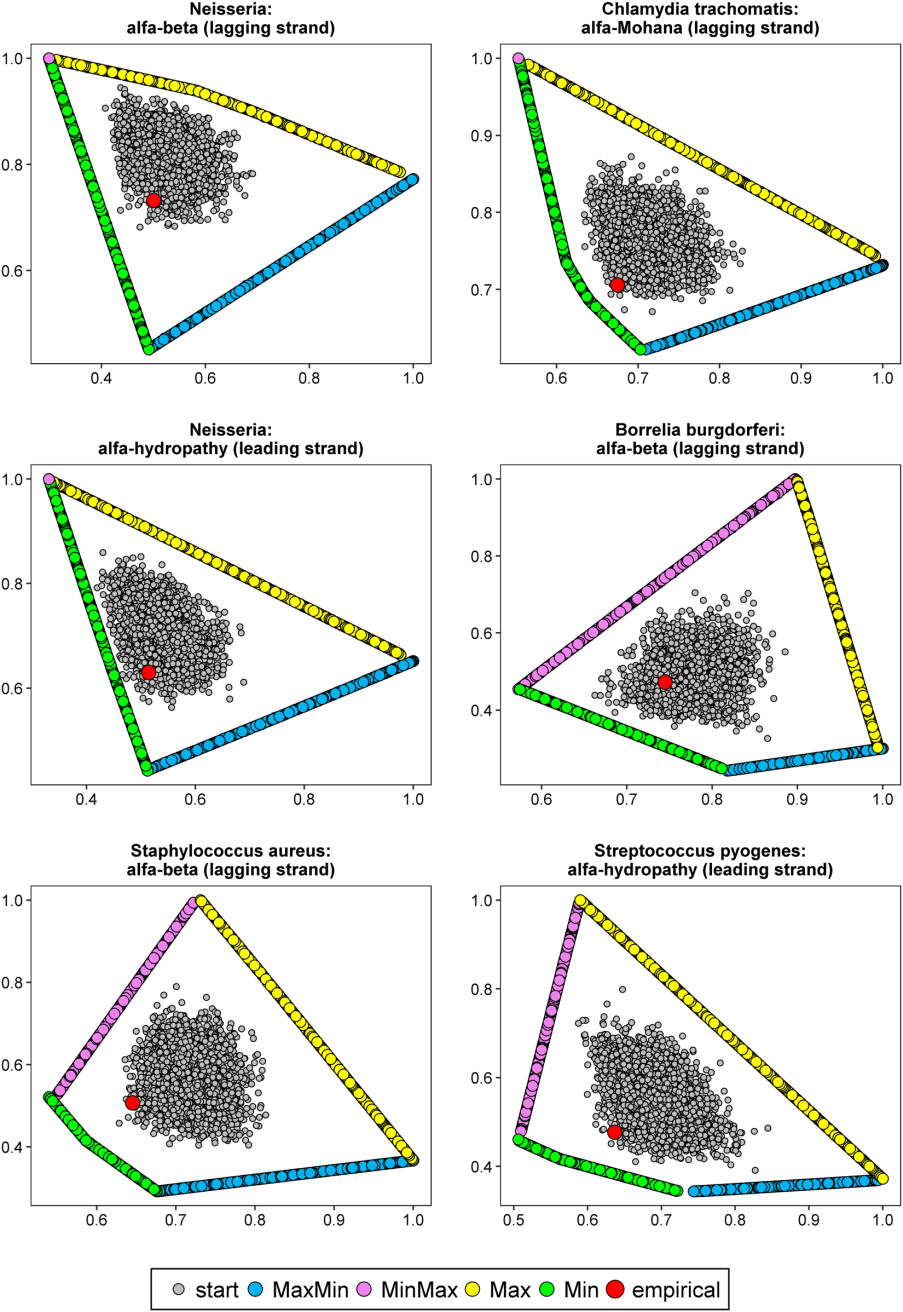
As in Fig. 4.

Examples of Pareto fronts of optimized matrices with starting and empirical matrices are presented in Figs 4 and 5. The shape of the fronts depends on pairs of compared properties. Optimized matrices create lines or curves, which converge to matrices from other scenarios represented by one point in the scale of the plots (Figs 4 and 5). Alternatively, lines representing all four possible types of optimized matrices create a polygon (Fig. 6). Nevertheless, in all cases starting and empirical matrices are surrounded by the Pareto fronts of the optimized matrices. The empirical matrices are placed usually at the edge of distribution of the starting matrices and close to the fronts of matrices that minimized at least one property.

There was no instance among 378 studied, in which an empirical matrix was located closest to the matrices maximizing two costs of amino acid replacements. In 96 cases, the relative minimal distances of empirical matrices was shortest to Pareto fronts of matrices minimizing two costs. For *Chlamydia muridarum*, there were 18 such cases, and 15 for *Chlamydia muridarum* and *Neisseria* (Table S2). Empirical matrices were usually closest to the Min Pareto fronts, when conformational parameter for alpha helix with beta-sheet and hydropathy indices as well as Grantham’s and Miyata’s matrices were considered (Table S3).

The relative average minimal distances of the empirical matrices to the Pareto fronts of matrices that minimized at least one parameter was significantly (p_BH_ < 0.0000001, Wilcoxon test) smaller (*r*_*MaxMin*&*MinMax*_ = 0.135, and *r*_*Min*_ = 0.204) than to matrices that maximized two costs (*r*_*Max*_ = 0.526). The average ratio of the minimal distances to Max and Min Pareto fronts *r*_*Max*_/*r*_*Min*_ was 3.4. The highest ratio showed empirical matrices from *Streptococcus* and *Bacillus* (>4) and the smallest from *Escherichia coli* (about 2) - Table S4. Empirical matrices were also about five times closer to minimizing than maximizing matrices, when were tested under the following physicochemical parameters: beta-sheet conformation with polarity and Mohana’s matrix as well as hydropathy with polarity and Mohana’s matrix (Table S5). The smallest ratio (about 2) was for pairs: alfa conformation with polarity and beta-sheet conformation as well as beta-sheet conformation with Grantham’s and Miyata’s matrices. We did not observe significant differences between performance of the matrices from the leading and lagging strands under the studied parameters.

### Comparison of matrices according to their structure and stationary distribution

To study the optimization level of empirical nucleotide matrices for hydropathy and polarity in relation to the structure of these matrices, we correlated the stationary frequencies of four nucleotides generated by these matrices with the ratio *r*_*Max*_/*r*_*Min*_, which measures the relative distance of the empirical matrices to the matrices that maximized and minimized the two costs. The analyses demonstrated a significant negative correlation between the adenine stationary frequency and the relative distance (Spearman correlation coefficient, ρ = −0.546, p-value = 0.019). It implies that the matrices that produce less adenine, minimize the costs of amino acid replacements more efficiently. Similar effectiveness was shown by the matrices that generate more cytosine. In this case, we observed significant positive correlation between the cytosine stationary frequency and the ratio *r*_*Max*_/*r*_*Min*_ (ρ = 0.494, p-value = 0.037). The stationary distribution of other nucleotides was not significantly correlated with *r*_*Max*_/*r*_*Min*_.

**Figure 7:**
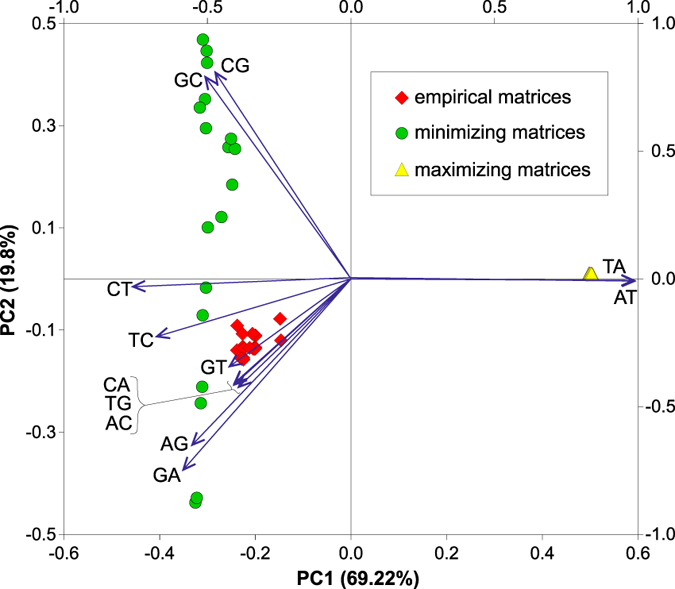
Biplot for results of Principal Component Analysis based on probability rates of the empirical matrices and matrices from Pareto fronts, which minimized or maximized both physicochemical costs of amino acid replacements A covariance matrix was assumed in the calculation of the principal components.

To further compare the empirical and optimized matrices according to their elements, i.e. transition probability rates, we calculated, for each case of genome and DNA strand, median values from the nucleotide substitution probability rates of matrices from Pareto fronts, which minimized or maximized both physicochemical costs of amino acid replacements (i.e. Min and Max scenarios, respectively). In Fig. S1, we compared distributions of the rates from these matrices. Moreover, to visualize and easy compare the matrices, we performed Principal Component Analysis on the 12 off-diagonal elements (Fig. 7). The first two principal components explained quite a lot of the total variance in the set, almost 90%. The empirical matrices create a cluster, which indicates that they are characterized by quite similar probability rates (Fig. 7). This group is very closely located to the matrices minimizing both costs and is very far from the maximizing matrices. The minimizing matrices are scattered according to the second component but are packed quite tightly in respect to the first component. The maximizing matrices are concentrated almost to one point in this scale, which implies very similar values of their probability rates.

The superposition of vectors representing variables (i.e. nucleotide substitutions) enabled to identify variables that contribute to the discrimination of the matrices. The maximizing matrices are characterized by larger values of substitution rates A → T and T → A than the empirical and minimizing matrices (see also Fig. S1). These substitutions are highly positively correlated with the first component (0.989 and 0.987, respectively). On the other hand, the empirical and minimizing matrices show greater probability of C → T and T → C substitutions, negatively correlated with the first component by coefficients −0.760 and −0.677, respectively. These four types of substitutions contribute most to the separation of the empirical and minimizing matrices from the maximizing ones.

The minimizing matrices are further differentiated in respect of two types of substitutions: C → G/G → C and A → G/G → A, which are correlated with the second component with coefficients: 0.808/0.791 and −0.651/−0.749, respectively. This differentiation is related with genome rather than DNA strand. The matrices that were minimized based on the stationary distribution of empirical matrices and codon frequencies from genomes of *Bacillus*, *Borrelia*, *Rickettsia*, *Staphylococcus* and *Streptococcus* are characterized by higher probabilities of C → G/G → C substitutions. In turn, greater values of A → G/G → A substitutions are typical of the minimizing matrices optimized on *Escherichia* and *Neisseria* genomes. Besides these transitions, the empirical matrices are also characterized by some increase of probability in substitutions of G → T, T → G, A → C and C → A (Fig. S1). The third principal component explained only 5.6% of variance and separated most minimizing matrices from several others including also empirical matrices. Substitutions C → T and G → T showed the largest correlation with this component (−0.605 and −0.561) and were more frequent in the second set of matrices.

Generally, both empirical and the minimized matrices are characterized by higher probability of substitutions A → G/G → A and C → T/T → C as well as lower probability of substitutions C → G/G → C than the maximized matrices (Fig. S1). Differences in these probabilities between the empirical/minimized and the maximized matrices are statistically significant (p < 0.05, Kruskal-Wallis test) but not between the empirical and minimized matrices (p > 0.38). The empirical and the minimized matrices show also significantly smaller probabilities of A → T and T → A substitutions than the maximized matrices. The empirical matrices differ from the both optimized matrices in larger values of A → C/C → A and G → T/T → G substitutions, whereas the minimized matrices have the highest probabilities of G → C/C → G substitutions of all matrices (Fig. S1). We obtained similar results when all 21 pairwise combinations of 7 physicochemical amino acid properties were analyzed (Fig. S2). Only the differences in G → T/T → G and G → C/C → G substitutions were less pronounced.

## Discussion

In this study, we checked to what extent the pattern of nucleotide substitutions in empirical mutational matrices from bacterial genomes minimizes or maximizes costs in replacements of amino acids. Since mutations are usually considered spontaneous and random, we could await no signs of this optimization. However, most mutations in protein-coding sequences are harmful, therefore, we can expect that mutational pressures should have a tendency to minimize their effect on protein genes. Since such types of sequences constitute a significant fraction of bacterial genomes, i.e. more than 90%^71^, it seems reasonable to analyze the optimization of mutation pressure in the context of these sequences. On the other hand, mutations are responsible for genetic variation of organisms, which accelerates their evolution and adaptation to variable environments. Then, an increased level of substitutions associated with positive selection should be expected. To verify these hypotheses, we compared the effect of the empirical matrices with reference matrices that were optimized to minimize and maximize costs of amino acid replacements according to physicochemical properties. In contrast to previous studies^63, 64^, we optimized the matrices simultaneously according to two properties and modelled the nucleotide substitutions by more general unrestricted model^72^ assuming only the same stationary distribution as the compared empirical matrices. Furthermore, the new model does not assume restrictions on the time-reversibility and the same convergence speed to the stationarity as the empirical matrices. Therefore, the optimized matrices were searched here in much larger space of possible solutions and the results have a more general significance.

The comparison of effects exerted by the matrices showed that the empirical matrices are quite well optimized to minimize the costs in amino acid replacements. Their influence on changes in physicochemical properties of amino acids was quite similar to the matrices that minimized costs in at least one of these two properties. Considering two extreme cases, in which costs in both properties were minimized or maximized, every empirical matrix was closer in the costs space to the matrices that minimised both criteria than matrices maximizing them. The results were independent on the genome and DNA strand (lagging or leading) from which the empirical matrix was derived. The empirical matrices appeared to minimize at best costs of amino acid replacement according to conformational parameter for alpha helix, which can related with the common prevalence of this secondary structure in proteins.

It should be emphasized that the obtained effect is not trivial because the studied empirical matrices were not disturbed by selection on the amino acid level and did not include the effect of selection associated with translational speed or accuracy^29, 73, 74^. It is also noteworthy that the matrices were evaluated based on changes in physicochemical properties of amino acids without influence of any mutational pressure. Thus the two studied components of the model are independent. Results would be not surprising if we considered matrices describing nucleotide substitutions accepted after selection in non-synonymous sites of protein-coding sequences and/or the effect of matrices was tested based on PAM amino acid matrices, e.g. Dayhoff, Blosum or JTT, which are derived from sequence comparisons and then include substitutions resulting from both mutation and selection.

Comparison of elements (i.e. probabilities of particular nucleotide substitutions) between matrices demonstrated that the studied minimization effects can be realized by decreasing probability of substitutions involving adenine and thymine. This observation is reflected in the genetic code, in which hydrophobic amino acids are coded by codons with thymine in the second position, whereas codons with adenine in such positions encode amino acids usually with hydrophilic properties^75–77^. As a result of this, the A↔T transversions in the second codon positions lead to drastic changes in properties of replaced amino acids. On the other hand, a higher probabilities of C↔T substitutions are associated with the matrices minimizing costs of amino acid replacements. Such features show also the empirical matrices. Interestingly, the C → T transition is a consequence of the spontaneous deamination of cytosine to uracil and its homologue 5-methylcytosine to thymine and belongs to one of the most frequent point mutations^3, 4, 6, 78, 79^.

Although the point mutations are consequences of spontaneous processes related with structure and properties of mutated nucleotides and nitrogenous bases, their rate and intensity can be modified during replication and repair processes. In the evolutionary scale, the variable nucleotide substitution rate can be accomplished by evolution of DNA polymerases with different fidelity introducing nucleotides during synthesis of new DNA strands^80–83^. Similarly, the evolution can be also subjected proofreading properties of polymerases^84, 85^ and other enzymes involved in post-replicative correction of mismatches^86, 87^. Besides the changes in the global mutation rate, also relative rates between nucleotide substitutions can be modified by differentiated preferences of polymerases and repairing enzymes for individual nucleotides^88–94^. The various pattern of nucleotide substitutions can be also associated with a fluctuating production and pools of individual nucleoside triphosphates, precursors of nucleotides incorporated during DNA replication^95–98^.

## Conclusions

Obtained results indicate that costs in amino acid replacements described by differences in their physicochemical properties and generated by bacterial mutational matrices are more similar to the matrices that minimized rather than maximized these costs. It implies that the empirical mutational matrices show a tendency to minimize consequences in amino acid replacements in products of protein-coding genes. The minimization is, however, not perfect because it is possible to find theoretical transition probability matrices that minimize costs more effectively than the empirical ones. Thereby, the empirical matrices can provide some genetic variation essential in adaptation of organisms to rapidly changing environments. Mutational pressures operating in bacterial genomes are not completely random and can be adjusted during evolution to current selective constraints. Thereby, the represent an interesting example of evolvability.

## Materials and Methods

### Empirical nucleotide substitution matrices

We studied empirical mutational pressures found in nine genomes represented different bacterial groups: *Bacillus*, *Borrelia burgdorferi*, *Escherichia coli*, *Chlamydia muridarum*, *Chlamydia trachomatis*, *Neisseria*, *Rickettsia*, *Staphylococcus aureus* and *Streptococcus pyogenes* ^29, 73, 74^ – Table S1. The pressures were expressed by mutational probability matrices describing all possible nucleotide point mutations. It should be noted that these matrices were derived from sequences subjected to neutral mutations in the absence of selection on amino acid properties, i.e. pseudogenes or synonymous sites in homologous genes of closely related species or strains. What is more, the authors eliminated highly expressed genes from the final set to get rid of a potential influence of selection associated with a specific codon bias and translational speed or accuracy^99–104^. Since bacterial genomes are characterized by significant mutational bias characteristic of differently replicated DNA strands^8, 15, 16, 105^, we analysed the mutational pressures for the leading and lagging strands, separately (see Table 2 for an example).

**Table 2:** The transition probability *P* matrix describing mutational pressure in the leading DNA strand from *Escherichia coli* genome.

	A	T	G	C
A	0.7600	0.0594	0.1394	0.0412
T	0.0452	0.7828	0.0508	0.1212
G	0.1534	0.0481	0.7720	0.0265
C	0.0368	0.2491	0.0290	0.6852

A nucleotide from the column is replaced by a nucleotide from the row.

### Generation of optimized nucleotide transition probability matrices

The empirical mutational matrices were compared with other transition probability matrices that were optimized according to costs of mutations. Each matrix describes a nucleotide substitution process. Mathematically speaking, it is a realization of continuous-time homogenous Markov process defined by a rate matrix *Q* = (*q*_*ij*_), where *q*_*ij*_ is a transition rate from nucleotide *i* to *j*. In this approach, we adopted the unrestricted (UNREST) model of nucleotide substitution^72^ presented in the Table 3.

**Table 3:** Substitution rate matrix *Q* for the unrestricted model of nucleotide substitutions (UNREST).

	A	T	G	C
A	—	*q*_*AT*_	*q*_*AG*_	*q*_*AC*_
T	*q*_*TA*_	—	*q*_*TG*_	*q*_*TC*_
G	*q*_*GA*_	*q*_*GT*_	—	*q*_*GC*_
C	*q*_*CA*_	*q*_*CT*_	*q*_*CG*_	—

The diagonals of *Q* are determined to each row sum up to 0. The nucleotide stationary distribution *π* = (*π*_*A*_, *π*_*T*_, *π*_*G*_, *π*_*C*_) is given by the set of equations *πQ* = 0 under the constraint ∑_*i*∈{*A*,*T*,*G*,*C*}_ *π*_*i*_ = 1.

This choice was due to the fact that it is the more general model than others commonly used (e.g. GTR). Therefore, this model can include more complex effects disregarded in the restricted models. Every UNREST-type rate matrix *Q* fulfils the following system of equations:

1 $$\pi Q=0,$$

where *π* = {*π*_*A*_, *π*_*T*_, *π*_*G*_, *π*_*C*_} is a stationary distribution of four nucleotides: adenine (A), thymine (T), guanine (G) and cytosine (C), without any extra assumption on *Q*. Since we were interested in the comparison of properties of the optimized matrices with the empirical mutational matrices, we assumed for the former the same stationary distribution as in the respective empirical matrices (Table 4).

**Table 4:** Nucleotide stationary distribution generated by matrices from leading and lagging DNA strands for studied genomes.

Genome	Leading strand				Lagging strand			
	A	T	G	C	A	T	G	C
*Bacillus* species	0.356	0.273	0.229	0.141	0.273	0.356	0.141	0.229
*Borrelia burgdorferi*	0.317	0.488	0.137	0.059	0.488	0.317	0.059	0.137
*Chlamydia muridarum*	0.245	0.252	0.282	0.222	0.225	0.227	0.290	0.259
*Chlamydia trachomatis*	0.234	0.214	0.293	0.260	0.253	0.252	0.253	0.242
*Escherichia coli*	0.247	0.328	0.247	0.179	0.268	0.308	0.207	0.217
*Neisseria* species	0.222	0.305	0.244	0.229	0.305	0.222	0.229	0.244
*Rickettsia* species	0.295	0.308	0.207	0.190	0.327	0.272	0.238	0.163
*Staphylococcus aureus*	0.407	0.393	0.121	0.080	0.353	0.450	0.087	0.110
*Streptococcus pyogenes*	0.326	0.420	0.123	0.131	0.301	0.402	0.094	0.203

To calculate the rates *q*_*ij*_ for the fixed stationary distribution *π*, we had to reformulate the system of equation (1). This procedure was described in details in Błażej *et al*.^106^. Briefly, this system of linear equation allows usually to find *π* for known rates but we wanted to calculate rates providing known *π*. Thereby, in the latter case *π* plays a role of coefficients, which leads to the following system of homogeneous linear equations:

2 $$X{\beta }^{T}=0,$$

where:

3 $$\beta =[{q}_{AT},{q}_{AG},{q}_{AC},{q}_{TA},{q}_{TG},{q}_{TC},{q}_{GA},{q}_{GT},{q}_{GC},{q}_{CA},{q}_{CT},{q}_{CG}]$$

and

4 $$X=[\begin{array}{cccccccccccc}-{\pi }_{A} & -{\pi }_{A} & -{\pi }_{A} & {\pi }_{T} & 0 & 0 & {\pi }_{G} & 0 & 0 & {\pi }_{C} & 0 & 0\\ {\pi }_{A} & 0 & 0 & -{\pi }_{T} & -{\pi }_{T} & -{\pi }_{T} & 0 & {\pi }_{G} & 0 & 0 & {\pi }_{C} & 0\\ 0 & {\pi }_{A} & 0 & 0 & {\pi }_{T} & 0 & -{\pi }_{G} & -{\pi }_{G} & -{\pi }_{G} & 0 & 0 & -{\pi }_{C}\end{array}]$$

under the general condition:

5 $$\mathop{\forall }\limits_{i\ne j}{q}_{ij} > 0.$$

We get immediately from linear algebra that every homogeneous linear system of equations has at least one trivial solution. If there is at least one nontrivial solution then infinitely many possible solutions exist. These solutions generate a vector space *V*, where operations are inherited from the finite-dimensional Euclidean space. Obviously, the equation (2) has at least one nontrivial solution. Therefore, we were able to find the set of nine linearly independent vectors (generators) *v*_1_, *v*_2_, …, *v*_9_ ∈ *R* ^12^ that span the vector space *V*. As a result, each considered stochastic process of nucleotide substitution with a given stationary distribution *π* has a unique representation:

6 $$\beta ={\beta }_{1}{v}_{1}+{\beta }_{2}{v}_{2}+\ldots +{\beta }_{8}{v}_{8}+{\beta }_{9}{v}_{9}$$

Clearly, the formula (6) is a linear combination of vectors *v*_1_, *v*_2_, …, *v*_8_, *v*_9_ with coefficients *β*_*i*_, *i* = 1, 2, …, 8, 9, whereas *β* is composed of rows of matrix *Q*. It is worth noting that from the condition (5) the rate matrices *Q* = (*q*_*ij*_) constitute only a subset (not a subspace) of the whole vector space *V*. This method allowed us to generate rate matrices *Q* under minimal restrictions.

Furthermore, we needed to transform every rate matrix *Q* to a transition probability matrix *P* = (*p*_*ij*_) because this representation was more appropriate in the context of calculating the fitness function. To do this transformation, we applied the uniformization method^107^, which is generally used to modify the original continuous-time Markov process with non-identical leaving rates *q*_*ij*_ to an equivalent of stochastic process, in which the transition epoch is generated by a suitable Poisson process with a fixed rate.

### Measure of fitness

To study the mutational effect of the empirical and optimized artificial matrices, we used an objective vector *F* consisting of two components describing costs of amino acid replacements and related with amino acid differences in two selected physicochemical properties (*a* and *b*), e.g. hydropathy and polarity:

7 $$F=({F}_{a},{F}_{b}),$$

where *F*_*i*_, *i* = *a*, *b* are costs of amino acid replacements in respective properties of amino acids:

8 $${F}_{i}=\sum _{ < c,d > \in D}u(c){p}_{c\to d}{g}_{i}(c,d),$$

where: *D* is the set of pairs of codons *c* an*d d*, which differ in one codon position, *u*(*c*) is the average usage of the codon *c* in respective protein-coding sequences, *p*_*c*→*d*_ is the probability of transition from the codon *c* to *d*, which is an element of a transition probability matrix *P*, whereas *g*_*i*_(*c*, *d*) is a difference between a physicochemical property of two amino acids which are coded by the codon *c* and *d*, respectively. The difference was based on several commonly used amino acid scoring matrices and indices describing various physicochemical and biochemical properties of amino acids: conformational parameter for alpha helix and beta-sheet^67^, hydropathy^65^, polarity^66^, Grantham’s chemical distance^68^, Miyata’s amino acid pair distance^69^ and Mohana’s EMPAR matrix^70^. In the case of indices, we calculated an absolute difference between the corresponding index values for given amino acids which are coded by the codon *c* and *d*. The matrices and indices were downloaded from AAindex database^108^. In the case when a sense codon was replaced into stop codon, we assumed their costs as the largest value of all amino acid substitution costs in the given measure.

To investigate simultaneously an optimization degree of mutational matrices according to the costs of change both in hydropathy and polarity, we applied a multiobjective optimization approach. In particular, we considered four scenarios of optimizing these costs for mutational matrices:

- Min, in which both costs were minimized;
- MaxMin, in which the cost of hydrophobicity change was maximized, whereas the polarity cost was minimized;
- MinMax, in which the cost of hydrophobicity was minimized, whereas the polarity cost was maximized;
- Max, in which both costs were maximized.

These criteria contain all possibilities in the optimization of two objectives.

The mutational effect exerted by empirical and optimized artificial matrices was investigated based on protein-coding sequences from bacterial genomes, for which the empirical mutational pressures were derived. The sequences and their annotations were downloaded from GenBank database^109^ – Table S1. Since differently replicated DNA strands in bacterial chromosomes are subjected to distinct mutational pressures, we considered the derived pressures and protein-coding sequences from the leading and lagging DNA strands separately. The location of these genes according to the DNA strands was deduced based on DNA asymmetry calculated in the Oriloc software^110^.

### Algorithm for finding optimized solutions

Evolutionary Multiobjective Optimization (EMO) approach is used in many optimization problems due to its simplicity and flexibility. Here, we used a modified version of the Strength Pareto Evolutionary Algorithm (SPEA2)^111^, which is an efficient technique used in many multiobjective optimization problems. Moreover, SPEA2 turned out to be very effective in comparison to others and has become one of the most important reference point in various recent investigations and applications, see e.g. Zitzler, *et al*.^112^. It produces a set of optimal solutions called Pareto set, which consists of non-dominated solutions such that none of their objective functions can be improved in value without degrading the other objective value. To visualize a tendency in solutions, we plotted Pareto fronts, which are sets of objective vectors calculated for elements from the respective Pareto sets.

**Figure 8:**
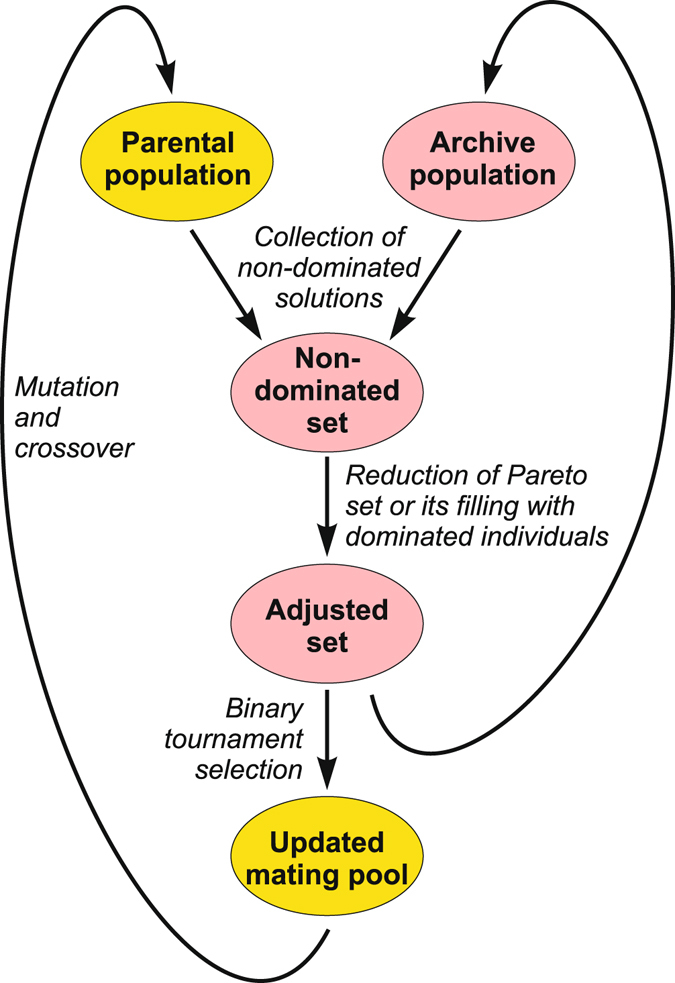
The workflow of the algorithm SPEA2.

The applied algorithm operates on potential solutions divided into parental (regular) and archive (external) populations (Fig. 8). The latter represents an approximation of the Pareto set and gathers the final solutions. In each optimized matrix case, the initial parental population consisted of 2000 randomly generated candidate solutions representing substitution probability matrices, which fulfilled conditions given by equations (4) and (5). After evaluation of fitness functions for individuals in the parental and archive populations, all their non-dominated solutions were copied into the new archive population. If its size exceeded the assumed limit of 500 individuals, the set was reduced by a truncation operator. Otherwise, the set was supplemented by the best dominated individuals from parental and archive populations. Next, in the mating selection stage, individuals from the new archive population were subjected to binary tournament with replacement to fill the mating pool. Winning individuals were mutated and recombined to increase variation in the set and then became the parental population for the next iteration step of this algorithm.

It is well known that every evolutionary based algorithm is founded on mutation and crossover operators, which are responsible for diversity of candidate solutions. Therefore, the most important step in using these algorithms is to define the structure of each candidate solution, which allows describing effectively the genetic operators. In our case, each candidate solution is uniquely defined by the formula (3). This representation implies directly the shape of mutation operator, which is defined as a random shift of *v*_*i*_ generated according to the Normal distribution *N*(0, *σ*). The final value of *σ* parameter was selected after its tuning during preliminary simulations tests. The crossover operator is a modified version of Linear Crossover LBGA^113^. It produces an offspring, which is a random linear combination of its parents. Obviously, we checked in every case the quality of newly produced offspring’s, i.e. if they possess a proper representation of mutational pressure, especially if they fulfil the condition given by the formula (5). It follows from the fact that both operators do not guarantee by themselves that this condition is hold.

The simulations run with the probability of mutation 0.9 and crossover 0.4 till 2000 steps. For each optimized matrix, we performed in total 21 independent runs, from which we collected the best optimized matrices, which were Pareto optimal under a given restriction for the objective vector *F* of fitness functions.

### Distance measure of empirical matrices to optimized matrices

To assess the effect of the empirical mutational matrices on costs of amino acid replacement in comparison to the optimized matrices, we calculated the minimal Euclidean distances *m*_*i*_, *i* = *Min*, *MaxMin*, *MinMax*, *Max* between costs produced by the empirical matrices and the respective artificial matrices lying on Pareto front that were optimized according to the four scenarios. Since the physicochemical indices were in different scales, the distance for the particular physicochemical property was normalized by the maximum cost found in all scenarios. Based on the normalized minimal distances, we calculated a relative minimal distance of empirical matrices to Pareto fronts for the individual scenarios *r*_*i*_, *i* = *Min*, *MaxMin*, *MinMax*, *Max*, which is defined by:

9 $${r}_{i}=\frac{{m}_{i}}{{m}_{Min}+{m}_{MaxMin}+{m}_{MinMax}+{m}_{Max}}$$

Clearly, *r*_*i*_ can be used as a quantitative measure of tendencies in optimization of costs in changes of amino acid properties by empirical matrices. This parameter has a value from 0 to 1. The small value implies that the costs produced by the empirical matrices are similar to those generated by matrices from the respective Pareto front (scenario).

## Electronic supplementary material

Supplementary information
