# Supplementary material for: Optimization of amino acid replacement costs by mutational pressure in bacterial genomes
Source: Sci Rep. 2017 Apr 21;7:1061. doi: 10.1038/s41598-017-01130-7 (PMC5430830; doi:10.1038/s41598-017-01130-7)

Supplementary information

Optimization of amino acid replacement costs by mutational pressure in bacterial genomes

Paweł Błażej, Dorota Mackiewicz, Małgorzata Grabińska, Małgorzata Wnętrzak, Paweł Mackiewicz

Table S1. Characteristics of genomes and their protein-coding genes used in the study.

Genome	Accession number	Number of genes on strand		Total gene length on strand [bp]	
		leading	lagging	leading	lagging
Bacillus cereus ATCC 10987, ATCC 14579, E33L, *B. anthracis* str. Ames; *B. thuringiensis* serovar konkukian str. 97-27	NC_003909, NC_004722, NC_006274, NC_003997, NC_005957	19,673	6,737	16,711,590	5,187,789
Borrelia burgdorferi B31	NC_001318	333	141	356,034	173,796
Chlamydia muridarum Nigg	NC_002620	512	407	539,778	436,323
Chlamydia trachomatis D/UW-3/CX	NC_000117	507	395	513,497	434,802
Escherichia coli str. K-12 substr. MG1655, O157:H7 str. Sakai, CFT073, 042	NC_000913, NC_002695, NC_004431, NC_017626	11,198	8,577	10,375,123	7,799,045
Neisseria gonorrhoeae FA 1090; *N. meningitidis* serogroup A strain Z2491, 8013, MC58, serogroup C FAM18	NC_002946, NC_003116, NC_017501, NC_003112, NC_008767	5,529	4,308	4,818,080	3,932,314
Rickettsia prowazekii Madrid E; *R. typhi* Wilmington; *R. felis* URRWXCal2; *R. conorii* Malish 7	NC_000963, NC_006142, NC_007109, NC_003103	2,688	1,764	2,364,246	1,588,938
Staphylococcus aureus N315, MRSA252, COL	NC_002745, NC_002952, NC_002951	5,908	1,933	5,497,047	1,547,097
Streptococcus pyogenes SF370, SSI-1, MGAS8232, MGAS6180, MGAS315, MGAS10394	NC_002737, NC_004606, NC_003485, NC_007296, NC_004070, NC_006086	8,834	2,193	7,895,787	1,803,942

Table S2. Number of cases, in which an empirical matrix from a genome was located closest to the given Pareto front of matrices maximizing one and minimizing other parameter (MaxMin and MinMax) as well as maximizing (Max) or minimizing (Min) both parameters.

Genome	Max	MaxMin	MinMax	Min
Bacillus species	0	15	15	12
Borrelia burgdorferi	0	12	29	1
Chlamydia muridarum	0	19	5	18
Chlamydia trachomatis	0	21	6	15
Escherichia coli	0	27	6	9
Neisseria species	0	20	7	15
Rickettsia species	0	13	21	8
Staphylococcus aureus	0	16	22	4
Streptococcus pyogenes	0	19	9	14

Table S3. Number of cases, in which an empirical matrix for a pair of amino acid properties was located closest to the given Pareto front of matrices maximizing one and minimizing other parameter (MaxMin and MinMax) as well as maximizing (Max) or minimizing (Min) both parameters.

Properties	Max	MaxMin	MinMax	Min
alfa-beta	0	0	2	16
alfa-Grantham	0	1	3	14
alfa-hydropathy	0	0	3	15
alfa-Miyata	0	0	3	15
alfa-Mohana	0	9	5	4
alfa-polarity	0	3	7	8
beta-Grantham	0	12	6	0
beta-hydropathy	0	17	1	0
beta-Miyata	0	13	5	0
beta-Mohana	0	7	5	6
beta-polarity	0	7	4	7
Grantham-hydropathy	0	1	17	0
Grantham-Miyata	0	0	18	0
Grantham-Mohana	0	14	4	0
Grantham-polarity	0	6	8	4
hydropathy-Miyata	0	17	1	0
hydropathy-Mohana	0	9	5	4
hydropathy-polarity	0	17	1	0
Miyata-Mohana	0	15	2	1
Miyata-polarity	0	10	6	2
Mohana-polarity	0	4	14	0

Table S4. Mean relative minimal distances of empirical matrices from bacterial genomes to respective Pareto fronts of matrices maximizing one and minimizing other parameter (MaxMin & MinMax) as well as maximizing (Max) or minimizing (Min) both parameters. Max/Min is an average ratio of the minimal distances to Max and Min Pareto fronts.

Genome	Max	MaxMin	MinMax	Min	Max/Min
Bacillus species	0.579	0.136	0.113	0.171	4.26
Borrelia burgdorferi	0.557	0.130	0.084	0.230	2.61
Chlamydia muridarum	0.493	0.118	0.201	0.188	3.55
Chlamydia trachomatis	0.475	0.115	0.197	0.213	3.23
Escherichia coli	0.405	0.107	0.186	0.302	1.82
Neisseria species	0.454	0.130	0.204	0.212	3.13
Rickettsia species	0.572	0.148	0.097	0.182	3.38
Staphylococcus aureus	0.608	0.124	0.091	0.177	3.77
Streptococcus pyogenes	0.590	0.112	0.141	0.157	4.49

Table S5. Mean relative minimal distances of empirical matrices for a pair of amino acid properties to respective Pareto fronts of matrices maximizing one and minimizing other parameter (MaxMin and MinMax) as well as maximizing (Max) or minimizing (Min) both parameters. Max/Min is an average ratio of the minimal distances to Max and Min Pareto fronts.

Properties	Max	MaxMin	MinMax	Min	Max/Min
alfa-beta	0.271	0.312	0.279	0.295	2.12
alfa-Grantham	0.339	0.275	0.245	0.260	2.59
alfa-hydropathy	0.282	0.285	0.312	0.299	2.61
alfa-Miyata	0.335	0.276	0.259	0.268	2.70
alfa-Mohana	0.393	0.134	0.267	0.200	2.67
alfa-polarity	0.277	0.192	0.325	0.259	1.93
beta-Grantham	0.535	0.064	0.086	0.075	2.03
beta-hydropathy	0.611	0.036	0.056	0.046	2.34
beta-Miyata	0.550	0.054	0.085	0.069	2.10
beta-Mohana	0.559	0.109	0.131	0.120	4.95
beta-polarity	0.593	0.119	0.139	0.129	5.54
Grantham-hydropathy	0.591	0.084	0.058	0.071	2.47
Grantham-Miyata	0.643	0.040	0.021	0.030	2.38
Grantham-Mohana	0.636	0.074	0.099	0.087	3.94
Grantham-polarity	0.599	0.107	0.121	0.114	4.12
hydropathy-Miyata	0.604	0.044	0.083	0.063	2.57
hydropathy-Mohana	0.626	0.082	0.110	0.096	4.87
hydropathy-polarity	0.652	0.075	0.124	0.100	4.96
Miyata-Mohana	0.650	0.070	0.091	0.081	4.31
Miyata-polarity	0.625	0.098	0.116	0.107	4.63
Mohana-polarity	0.669	0.085	0.058	0.071	4.72

Fig. S1. Nucleotide substitution rates for empirical matrices (emp) as well as matrices that minimized (min) and maximized (max) hydropathy and polarity costs of amino acid replacements, separately for leading (lead) and lagging (lagg) strands.. The thick horizontal lines indicate median, the coloured boxes show quartile range and the whiskers determine the range without outliers.

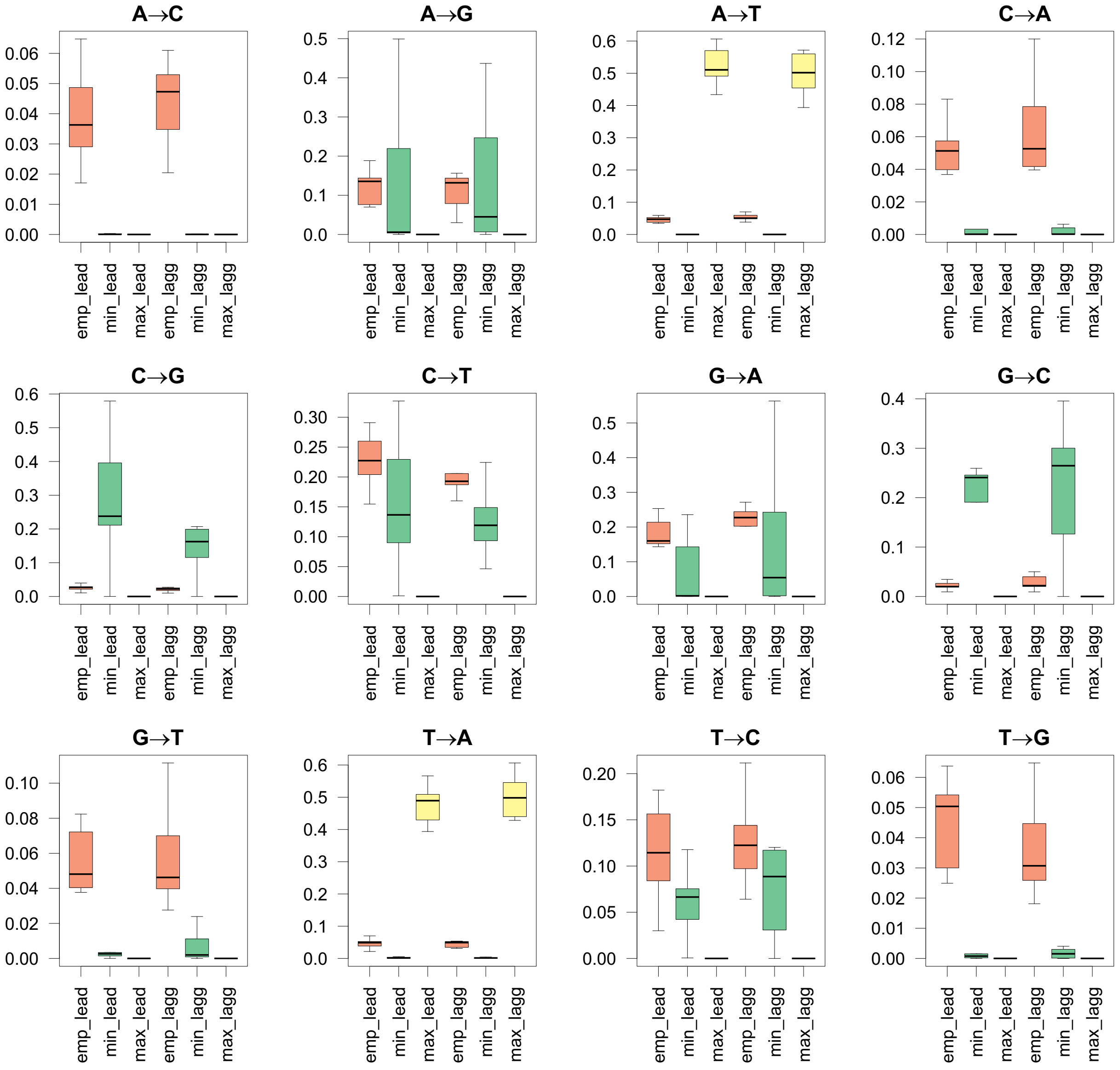

Fig. S2. Nucleotide substitution rates for empirical matrices (emp) as well as matrices that minimized (min) and maximized (max) various physicochemical costs of amino acid replacements, separately for leading (lead) and lagging (lagg) strands.. The thick horizontal lines indicate median, the coloured boxes show quartile range and the whiskers determine the range without outliers.

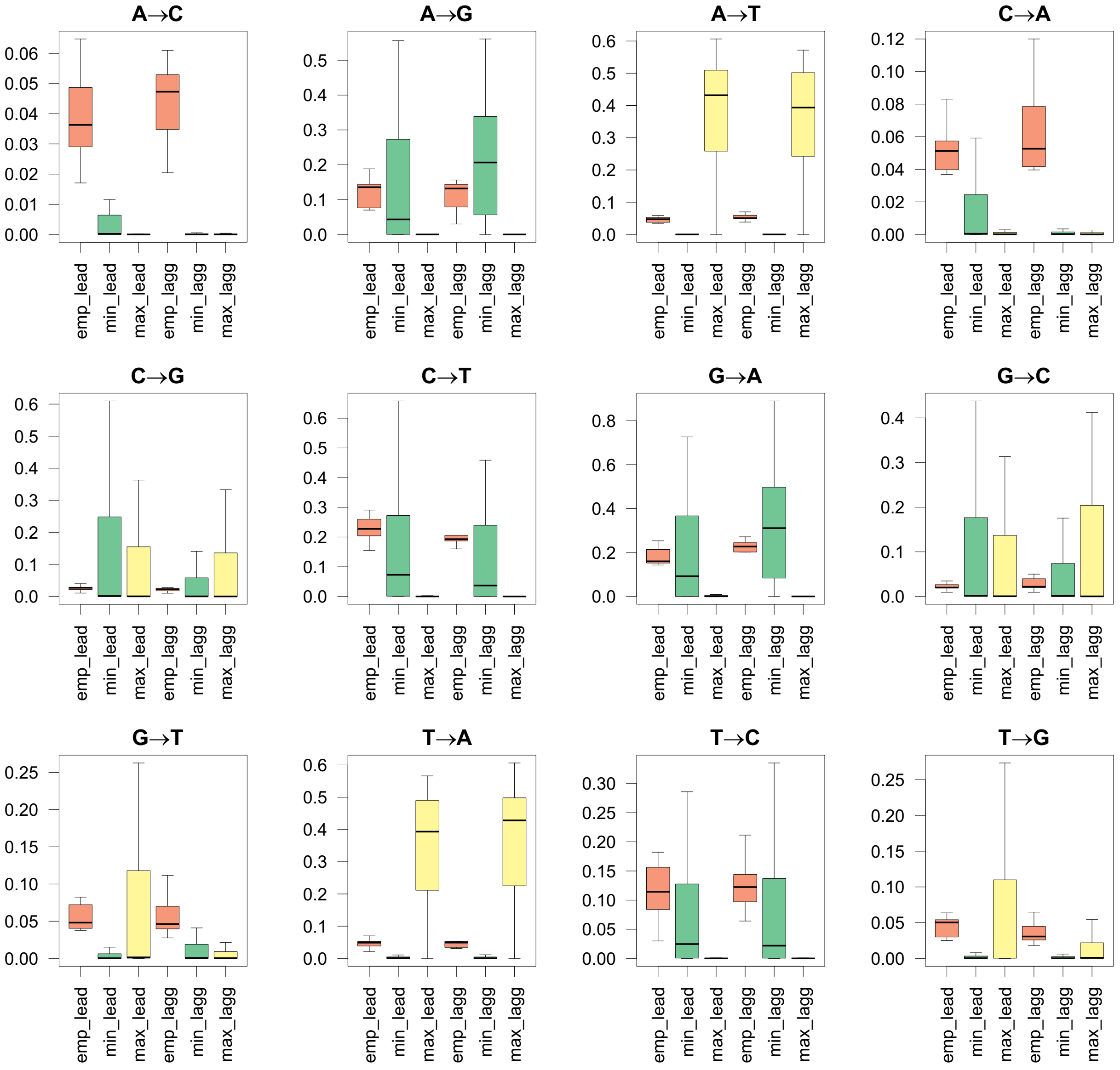

Supplement: Supplementary file 1 — Supplementary information [file 41598_2017_1130_MOESM1_ESM.pdf]
